# Solid‐phase silica‐based extraction leads to underestimation of residual DNA in decellularized tissues

**DOI:** 10.1111/xen.12643

**Published:** 2020-09-15

**Authors:** Tara C. Schmitz, Aysegul Dede Eren, Janne Spierings, Jan de Boer, Keita Ito, Jasper Foolen

**Affiliations:** ^1^ Orthopaedic Biomechanics Department of Biomedical Engineering Eindhoven University of Technology Eindhoven The Netherlands; ^2^ BioInterface Science Department of Biomedical Engineering Eindhoven University of Technology Eindhoven The Netherlands; ^3^ Institute for Complex Molecular Systems Eindhoven University of Technology Eindhoven The Netherlands

**Keywords:** decellularization, DNA quantification, tissue engineering, underestimation, xenotransplantation

## Abstract

Decellularization of animal tissues is a novel route to obtain biomaterials for use in tissue engineering and organ transplantation. Successful decellularization is required as animal DNA causes inflammatory reactions and contains endogenous retroviruses, which could be transmitted to the patient. One of the criteria for successful decellularization is digestion (fragmentation) and elimination (residual quantity) of DNA from the tissue. Quantification of DNA can be done in many ways, but it has recently been shown that silica‐based solid‐phase extraction methods often do not completely purify in particular small DNA fragments. In the context of decellularization, this means that the measured DNA amount is underestimated, which could compromise safety of the processed tissue for in‐patient use. In this article, we review DNA quantification methods used by researchers and assess their influence on the reported DNA contents after decellularization. We find that underestimation of residual DNA amount after silica‐based solid‐phase extraction may be as large as a factor of ten. We therefore recommend a direct assessment of DNA amount in tissue lysate using dsDNA‐specific binding dyes, such as Picogreen, due to their higher accuracy for small fragment detection as well as ease of use and widespread availability.

## 1 INTRODUCTION

In times of global population growth and an increased lifespan, the demand for organs and tissue‐based regenerative strategies is ever increasing.^1^ Due to a shortage of available organ donors, usage of animal tissues (xenografts) has emerged as an alternative for transplantation and scaffold‐based tissue engineering.^2^, ^3^ However, xenografts need to be processed to prevent graft rejection and inflammatory reactions.^3^ For this, removal of surface antigens as well as animal DNA from the source tissue is required, a process called decellularization. While surface antigens may lead to hyperacute graft rejection,^4^ DNA from animals harbors endogenous retroviruses that may be transmitted to patients.^5^ Additionally, extracellular DNA is known to cause inflammatory reactions *via* several signaling pathways, necessitating DNA‐removal from the tissue.^6^, ^7^ Consequently, accurate measurements are needed to detect short fragmented DNA (sfDNA) remaining in decellularized tissues. However, processing of decellularized samples for DNA quantification may affect the results of such quantification, depending on the method. In the following paragraphs, common methods for DNA extraction are explained in more detail.

**FIGURE 1:**
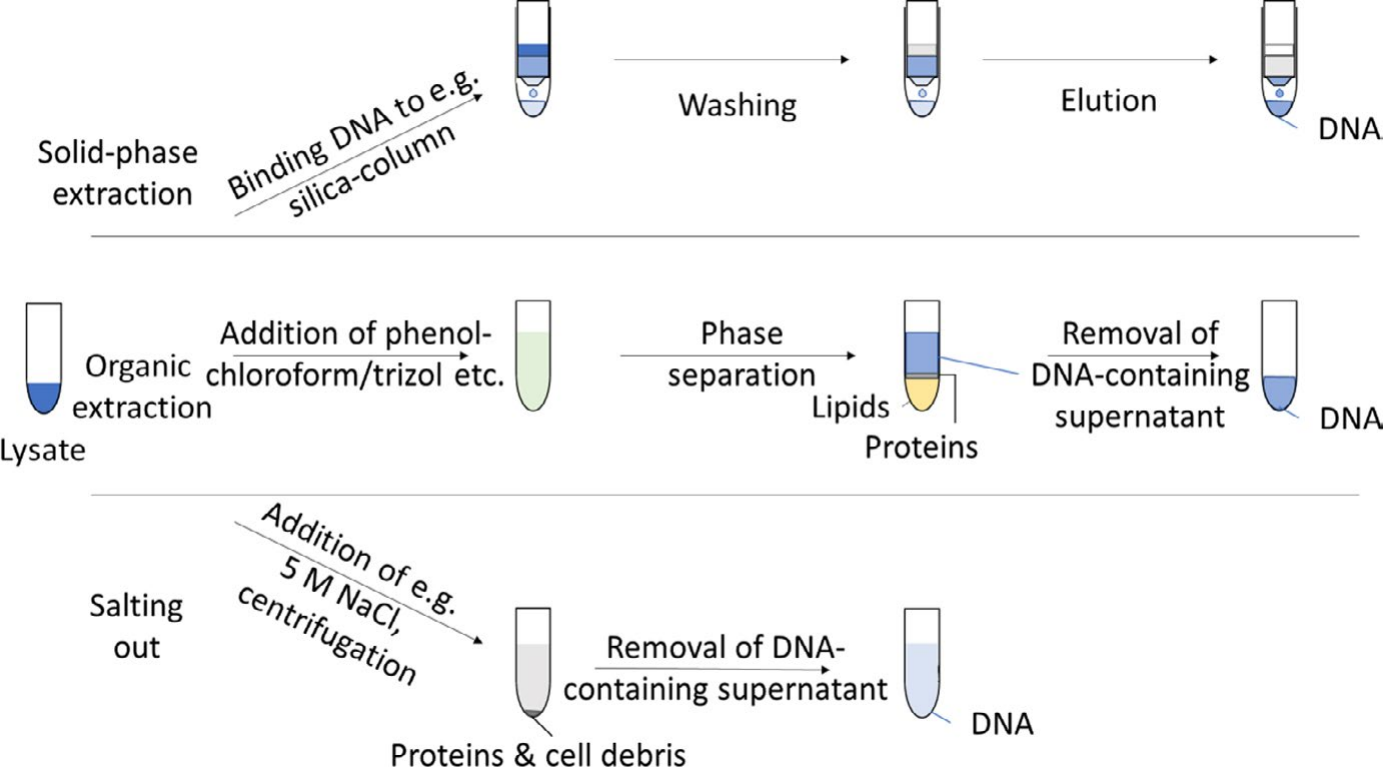
Overview of common DNA extraction protocols used on tissue lysate

Before the amount of residual DNA in decellularized tissues can be assessed, the tissue is digested, the cells are lysed (if any remain), and the DNA is dissolved in the buffer solution. Thereafter, the amount of DNA can be directly assessed by adding a fluorescent probe to the digested sample. Alternatively, the DNA can be further purified from the sample using an extraction procedure (Figure 1), exploiting its physicochemical properties. Phenol/chloroform‐extraction of DNA exploits differences in solubility of DNA vs. proteins and lipids in water‐/oil‐based solvents, respectively. A mixture of 25:24:1 phenol/chloroform/isoamyl alcohol is added to the sample, which is then vortexed for emulsification before centrifugation to ensure phase separation. While lipids are dissolved in the organic phase, proteins remain at the interphase and DNA in the aqueous supernatant, which can be transferred into a new vessel for quantification and further analysis. ^8^, ^9^

Alternatively, high concentrations of salt can be used to precipitate proteins and cellular debris, due to their hydrophobicity, while the DNA remains in the supernatant. This method is often preferred over the phenol/chloroform‐based extraction, as is does not rely on hazardous chemicals.^8^, ^10^

Solid‐phase extraction exploits interactions of DNA with a solid substrate, such as silica resin/beads in the presence of chaotropic salts, allowing for rapid purification of DNA from digested samples. Immobilization of DNA to the silica‐surface is based on electrostatic interactions, only allowing for release in the presence of hypotonic buffers. Especially for sfDNA, however, this does not recover the total amount. Investigations into the recovery of sfDNA from solid‐phase extraction kits have shown that for DNA fragments < 50 bp and < 100 bp, only about 16.5% and 27.7% (median across various extraction kits) are recovered, respectively.^11^, ^12^, ^13^

In the context of decellularized tissues, the method chosen for sfDNA extraction therefore biases the interpretation of results. This has subsequent consequences for suitability for in‐patient applications due to possible immunological side‐effects. Here, we review DNA extraction methods used in decellularization studies, discuss their effect on clinically safe use and identify suitable methods for DNA quantification in decellularized tissues.

## 2 RESULTS AND DISCUSSION

**FIGURE 2:**
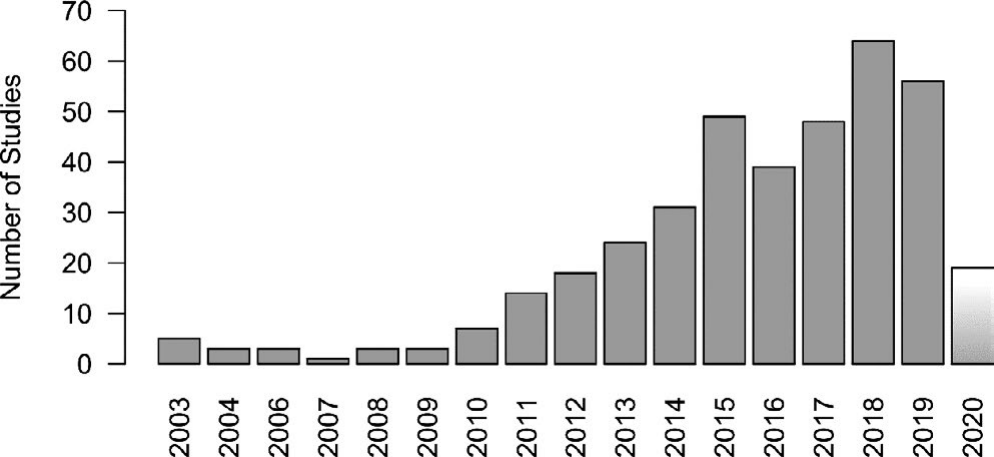
Increasing number of decellularization protocols published over the last 20 years. Note that PubMed was searched for decellularization studies in March 2020, that is, the 2020 bar doesn't represent the full year. 387 studies were identified in total

For this study, PubMed was searched for papers on decellularization methods for tissues by searching for “decellulariz*” OR “decellularis*” in title/abstract AND “DNA” as text word (see Appendix S1). 387 publications were reviewed for their DNA quantification approach for decellularized tissues. Over the past 20 years, a clear trend can be seen with an ever‐increasing number of publications describing protocols for decellularization of various tissues and uses thereof in regenerative medicine (Figure 2). Reducing the amount of residual DNA in these decellularized tissues is crucial for their further clinical application.

**FIGURE 3:**
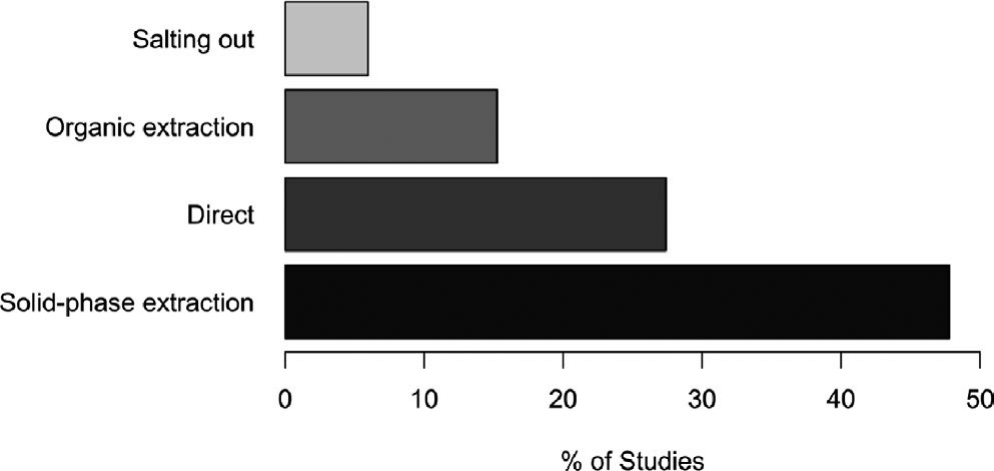
(Non‐)extracting DNA methods prior to quantification. Of the 387 identified studies, 186 employ solid‐phase‐based DNA extraction (93% of these solid phases are silica‐based), 106 quantify DNA directly in tissue lysate, 59 perform organic extraction of DNA, while 23 studies utilize salting‐out protocols protocols (note that some studies used several methods). 21 studies did not specify the quantification method

A large portion of research groups quantify residual DNA levels after extracting the DNA from the tissue lysate (ca. 70%), with a steady popularity of spin‐column silica‐based solid‐phase extraction (Figure 3). All in all, ≈50% of the conducted decellularization studies extract DNA *via* solid‐phase adsorption prior to quantification, while 15% use an organic extraction protocol and 5% extract DNA *via* salting‐out protocols. A consistent majority of research groups rely on silica‐based DNA extraction for quantification of residual DNA, while a minority relies on specific interactions between DNA‐binding dye facilitating direct detection in crude tissue lysates.

Recently, emerging evidence suggests that commercially available solid silica phase DNA extraction kits do not recover small dsDNA‐fragments from solution, as typically found in decellularized tissue.^11^ This underestimates the amount of measured residual DNA, which can thus impact the clinical usability. DNA interacts with silica electrostatically and hydrophobically *via* the negatively charged backbone (phosphate‐ions) and positively charged silica.^14^ This means that there are more DNA‐resin‐bonds found for large DNA‐molecules than small ones. Small fragments can consequently get lost in subsequent washing steps^15^ due to the applied shear forces from centrifugation/pipetting that rupture the bonds.

**FIGURE 4:**
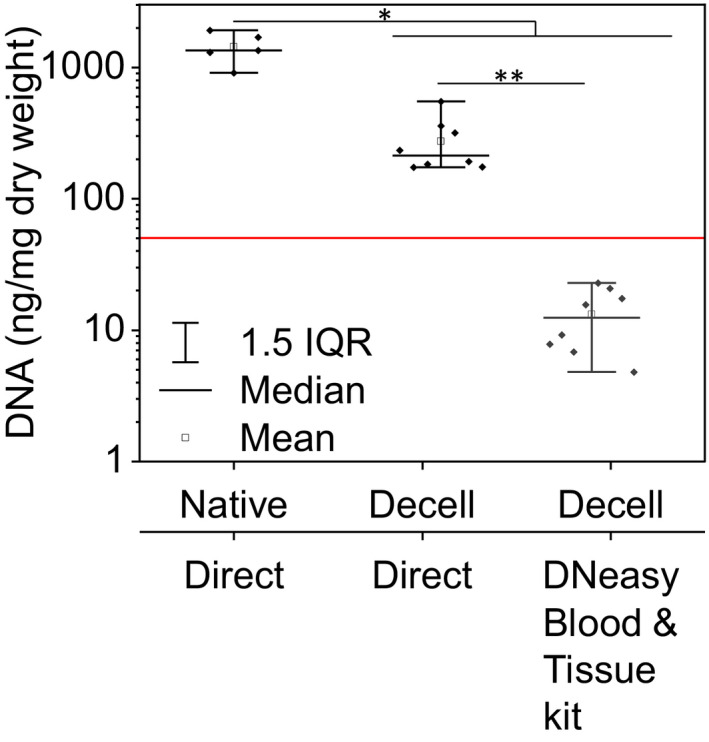
Silica‐based solid‐phase extraction of DNA from digested decellularized anterior cruciate ligament samples severly depletes DNA before quantification. Porcine anterior cruciate ligament (ACL) was decellularized based on a previously published protocol employing freeze‐thaw cycles, washes in detergent or ultrapure water, and enzymatic digestion of DNA (see Appendix S1).^53^ Samples were then handled either according to the DNeasy Blood & Tissue kit (Qiagen, Venlo, Netherlands), or digested overnight at 60°C using 140 mg/mL papain (Sigma‐Aldrich, Zwijndrecht, Netherlands) prior to DNA quantification using the Qubit platform (Invitrogen, Fisher Scientific, Landsmeer, Netherlands). More information available in Appendix S1. Native sample n = 5, decellularized samples n = 8. Values for remaining DNA in anterior cruciate ligaments across different quantification groups stem from the same samples. Statistical differences were investigated with a pairwise Wilcoxon test, assuming *P* < .05 as a significant difference between groups. * *P* < .05, ** *P* < .01. Red‐line marks the 50 ng/mg dry weight recommended limit.^35^

The DNA fragment size distribution in decellularized samples is dependent on the chosen decellularization protocol, enzyme concentration, and incubation time. Assuming optimization of DNA digestive conditions, the problem of DNA underestimation just becomes exacerbated, as fragments will become increasingly smaller, making them harder to accurately quantify after silica‐based solid‐phase extraction methods. Based on the median relative recovery of fragments < 200 bp estimated from Cook *et al*, this would lead to a gross underestimation of DNA content. Results from Tsai *et al* and our group (Figure 4) suggest that obtained values for sfDNA may differ by as much as 10‐fold.^13^ This effect may even be increased in the presence of ceramics,^16^ but may be less pronounced for perfusion‐based decellularization processes flushing out small DNA fragments, thus reducing the potential underestimation by DNA extraction.

Modifying the surface structure of the solid phase used, may improve sfDNA recovery, however, the investigated fragment sizes are often still > 100 bp and the studies’ results might not necessarily apply to even smaller fragments of DNA as found in decellularized tissues.^17^, ^18^ One study investigated sfDNA adsorption onto silica‐coated magnetite particles, achieving ≈54% adsorption of available sfDNA (80‐160 bp). ^19^ To the best of our knowledge, none of these modified substrates are readily commercially available. Careful adjustment of buffer‐conditions may increase yield of fragments ≥ 20 bp, but are not discussed in commercially available kits and therefore not routinely used.^20^ Complicating the situation is the lack of available information on the smallest extractable sfDNA using commercially available extraction kits (Table 1). Most groups use silica‐membrane‐based extraction kits, optimized for genomic DNA extraction from tissues, that is, optimized for large DNA fragment recovery. Moreover, these kits often do not state a lower limit of extraction concerning DNA fragment sizes. This highlights the need for either determining and correcting for the relative loss of sfDNA prior to using commercially available kits, or employing extraction methods that are more suitable for small fragment recovery.

**TABLE 1:** Solid‐phase DNA extraction kits used by research groups for extraction of DNA from (decellularized) tissues

Used kit (Supplier)	Studies		Lower DNA fragment size extraction limit	Usual size range of extracted DNA fragments
	No.	%		
AccuPrep®, Genomic DNA Extraction Kit (Bioneer)^a^	1	0.54	Not stated	Not stated
AllPrep DNA/ RNA Mini Kit (Qiagen)	1	0.54	Not stated	15‐30 kbp
Favorprep™ Tissue Genomic DNA Extraction Mini Kit (Favorgen)	1	0.54	Not stated	not stated
Genomic‐tip 500/G (Qiagen)^2^	1	0.54	Not stated	20‐150 kbp
Genomic DNA isolation kit (DENAzist)	1	0.54	Not stated	Not stated
Illustra™ Tissue and Cells Genomic Prep Mini Spin Kit (GE Healthcare)	1	0.54	>20 kbp	Not stated
Invisorb Spin Tissue Midi Kit (Invitek)	1	0.54	>180 bp	Not stated
LaboPass Tissue DNA Purification Kit (Hokkaido System Science Co. Ltd.)	1	0.54	Not stated	Not stated
peqGOLD Tissue DNA Mini Kit (peqlab)	1	0.54	Not stated	Not stated
PrimePrep Genomic DNA Isolation Kit (Genet Bio)	1	0.54	Not stated	Not stated
G‐spin™ Total DNA Extraction Kit (iNtRON Biotechnology)	1	0.54	Not stated	20‐30 kbp
UltraClean tissue and cell DNA isolation kit (Mo Bio Laboratories)	1	0.54	Not stated	Not stated
Isolate II Genomic DNA Kit (Bioline GmbH)	2	1.08	Not stated	Not stated
NucleoSpin kit (Macherey‐Nagel)	2	1.08	Not stated	Not stated
QIAamp DNA FFPE tissue kit (Qiagen)	2	1.08	Not stated	Not stated
ReliaPrep™ gDNA Tissue Miniprep System (Promega)	2	1.08	Not stated	Not stated
GeneJet DNA purification kit (Thermo Scientific)	4	2.15	>30 kbp	Not stated
GenElute mammalian genomic DNA miniprep kit (Sigma‐Aldrich)	8	4.30	Not stated	Not stated
TIANamp Genomic DNA assay kit (Tiangen Biotech)	9	4.84	Not stated	Not stated
PureLink® Genomic DNA Mini Kit (Invitrogen)	14	7.53	Not stated	20‐50 kbp
QIAamp DNA kit (Qiagen)	25	13.44	Not stated	20‐30 kbp
DNeasy Blood & Tissue Kit (Qiagen)	97	52.15	>100 bp	≈30 kbp
Unspecified	9	4.84	–	–

^a^ Note that this kit works with glass fiber‐based solid phase.

^b^ Note that this kit works with a diethylaminoethanol‐based anion exchange resin.

Our search did not result in studies examining potential bias toward certain DNA fragment sizes based on their solubility in presence of high ion‐concentrations. The suitability of salting‐out proteins for purification of DNA is therefore difficult to judge. From a usability standpoint, while organic extraction protocols do seem to enable sfDNA recovery,^13^ they employ hazardous chemicals unfit for large‐scale and routine use, while salt precipitation can be accomplished with non‐hazardous chemicals like NaCl.

Typically, DNA concentration after extraction and purification is measured spectrophotometrically, assessing the absorption value at 260 nm. Alternatively, colorimetric quantitation is used, and more sensitive in the sub‐µg range.^21^ A different approach utilizes quantitative real‐time PCR for DNA quantification, of which the reproducibility is however dependent on the initial DNA extraction method chosen, as well as potential interference from non‐DNA components in the sample itself.^12^, ^22^

Probably easiest is the direct quantification of DNA in digested tissue samples. This has obvious consequences for the detection method, as other tissue components and the homogeneity of the lysate will affect spectrophotometric approaches. Addition of a fluorophore, however, has been demonstrated to be a highly sensitive and reproducible approach for DNA detection in whole blood, serum, urine, and in the presence of proteins^23^ and glycosaminoglycans.^24^ Especially sensitive for detection of DNA in low amounts are PicoGreen and SYBR Green, contrary to ethidium bromide and Hoechst‐based dyes.^25^ The binding site sizes of all probes are smaller than the DNA fragments produced by commonly used DNases used in decellularization protocols (eg, Benzonase cleaves DNA to fragments of ca. 5 bp in size,^26^ while DNase I leaves fragments of ≥ 10 bp size^27^), enabling them to detect even small fragments to varying degrees. Although these fluorescent dyes exhibit sequence‐dependent specificity, with Hoechst and SYBR Green preferentially binding to AT‐rich sequences whereas PicoGreen binds more often to GC‐rich regions,^28^, ^29^, ^30^, ^31^, ^32^, ^33^ this effect is most likely negligible in the context of whole (cleaved) genome detection. More important is the use of an appropriate control sample of sfDNA that exhibits similar fragment size compared with samples obtained from decellularized tissues to account for differences in dye saturation of small versus large DNA fragments.^34^ There are several commercial kits available utilizing dsDNA‐binding fluorophores like Picogreen with high specificity. Some of these are designed in a 96‐well format, that is, enabling high‐throughput testing.

An often‐cited limit for acceptable DNA levels is 50 ng/mg dry weight of decellularized tissue with <200 bp in fragment length.^35^ The fragment length limit is derived from the smallest generally observed fragment length in apoptosis and extracellular DNA length in healthy individuals.^36^ Investigating the fragment size distribution of dilute DNA can be performed after concentrating the residual DNA from the tissue lysate. Usage of centrifugal filters for this purpose is quick, easy, and reliable. These filters are also used in cleanup of PCR‐products and specific retention of DNA fragments based on their size. We propose that a molecular weight cut‐off of <30 kDa is suitable for concentration of DNA from decellularized, digested tissue samples for subsequent gel electrophoresis.^37^

The origins of the acceptable absolute amount to define “decellularization” are somewhat nebulous. So far, studies on extracellular cell‐free DNA mostly focus on its abundance in serum or plasma, where it functions as a reporter of various diseases.^38^ Also here, the use of (non‐)extracting approaches to DNA quantification results in vastly different reported values.^39^ Perhaps, a better indicator of an acceptable DNA limit can be derived from the amount of tissue‐dependent apoptosis. Clearance of cellular debris, including fragmented DNA, is crucial for functioning tissue homeostasis.^40^ If too much DNA resides in the xenograft, this internal clearance system may get overwhelmed, and remaining extracellular DNA may then lead to inflammation. Thus, determining the cell number/mg of target tissue (assuming ≈7 pg DNA/cell ^41^) as well as the percentage of apoptotic cells allows for an estimate of permissible DNA levels in xenografts at the site in question.

**FIGURE 5:**
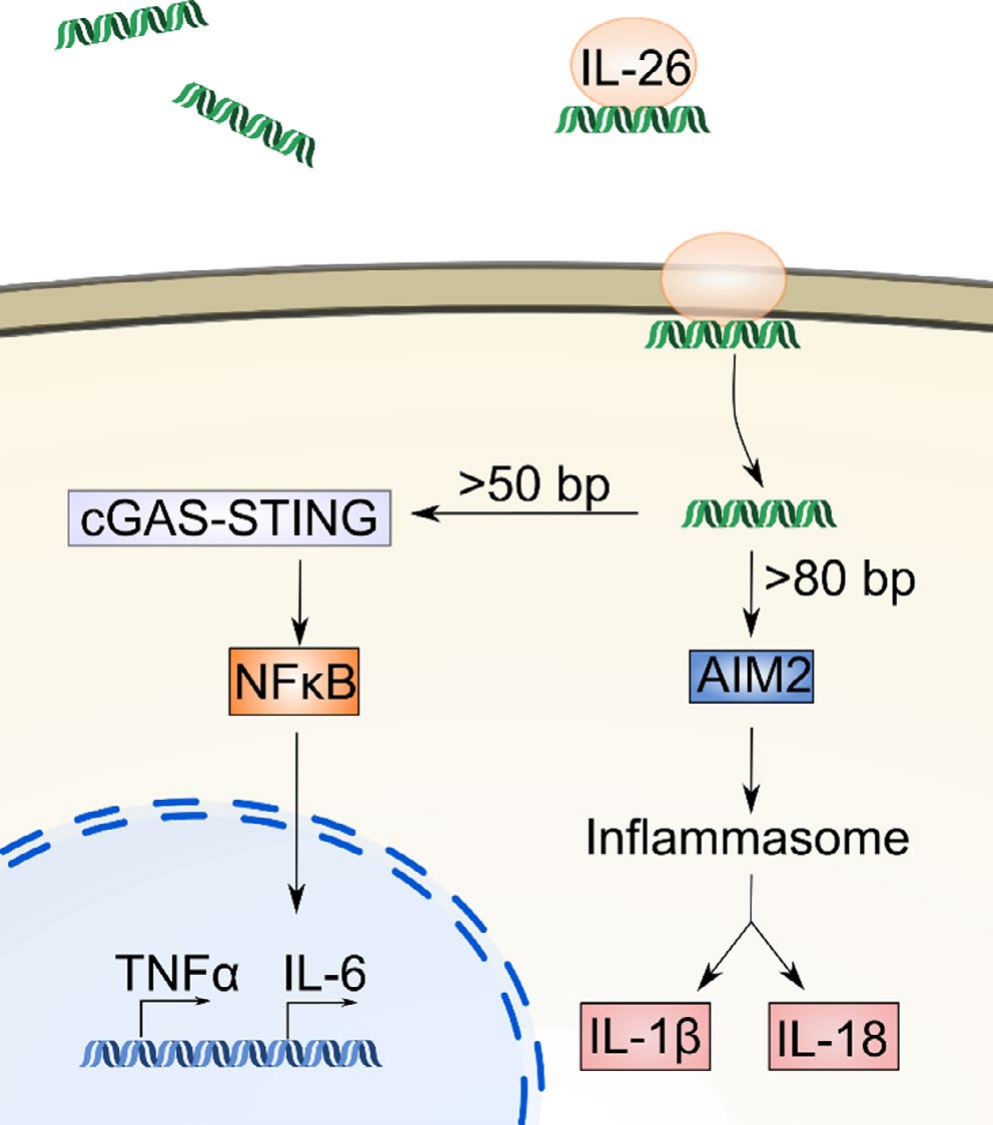
Proposed inflammatory signaling pathways in response to high amounts of uncleaved extracellular DNA from xenografts

Currently, there are no published systematic studies investigating the relation between residual DNA amount, fragment length, and immunological reaction in vivo, following xenotransplantation. A key player in extracellular dsDNA‐recognition and downstream signaling is interleukin 26 (IL‐26; Figure 5).^42^ The binding site size for DNA on IL‐26 is not defined yet; however, a minimal fragment size of >6 bp for DNA to be bound is expected, based on the predicted recognition site and the biophysical structure of amino acid α‐helices as well as DNA.^43^, ^44^ Recognition of non‐self DNA *via* IL‐26 and subsequent transfer into the cell has been tied to cyclic GMP‐AMP synthase and Stimulator of Interferon Genes (cGAS‐STING)‐mediated inflammation in myeloid cells, which ultimately leads to production of TNFα, and IL‐1β and IL‐6 activation.^42^, ^45^ Once in the cytosol, another signaling pathway including the Absent In Melanoma 2 (AIM2) protein recognizing the DNA ultimately leads to activation of the inflammasome and downstream maturation of IL‐1β and IL‐18.^45^ cGAS‐STING and AIM2 bind to DNA of >50 and >80 bp, respectively, reflecting the need for accurate determination of sfDNA content in decellularized tissues.^46^, ^47^, ^48^, ^49^, ^50^, ^51^, ^52^

## 3 CONCLUSION

Immunological sensing of DNA is one possible adverse reaction to xenotransplants in vivo. Accurate determination of DNA amount and fragment size distribution is therefore paramount in assessing the clinical suitability of decellularized tissues. From the currently available facts, DNA extraction from decellularized tissues *via* silica‐based approaches is not advisable due to depletion of sfDNA, leading to an underestimation of total DNA content. More suitable are solvent‐based extraction methods utilizing, for example, phenol/chloroform, or methods selectively precipitating proteins and cell debris for DNA isolation. Alternatively, direct assessment of DNA in tissue lysate can be performed. As no extraction procedure is performed, no bias in DNA detection is given, and the obtained value is expected to more accurately reflect residual DNA in the sample.

## CONFLICTS OF INTEREST

The authors declare no conflicts of interest.

## Supporting information

Appendix S1
