# Supplementary material for: Solid‐phase silica‐based extraction leads to underestimation of residual DNA in decellularized tissues
Source: Xenotransplantation. 2020 Sep 15;28(1):e12643. doi: 10.1111/xen.12643 (PMC9286341; doi:10.1111/xen.12643)
Supplement: Supplementary file 1 — Appendix S1 [file XEN-28-0-s001.docx]

# Methods

## Literature review

PubMed was searched for papers on decellularization methods for tissues by searching for “decellulariz*” OR “decellularis*” in title/abstract AND “DNA” as text word. Only peer-reviewed non-review articles with available full text were considered. Articles not quantifying the DNA amount after decellularization (18 articles) or using only semiquantitative methods/not quantifying DNA at all (15 articles) were further excluded. Three articles were excluded due to lack of decellularization of a tissue. Ten reviews and six off-topic articles were deselected. For six articles, no full text could be obtained. One retracted article and one paper from conference proceedings were further excluded. Finally, 387 articles were identified (as of March 2020) and analyzed for their DNA quantification strategy.

## Tissue decellularization

Porcine anterior cruciate ligament (ACL) was decellularized based on a shortened previously published protocol.^1^ Briefly, samples were subjected to 5 freeze-thaw cycles (2 min in liquid N_2_, 10 min in 37°C in ultrapure water) prior to incubation for 24 h in ultrapure water. Samples were then submerged in 1% Triton X-100 in Tris-EDTA (pH 7.6) (Sigma Aldrich, Zwijndrecht, Netherlands) for 24 h, washed for 2 min in ultrapure water and then digested with 25 U/ml Benzonase (Merck, Schiphol-Rijk, Netherlands) in 50mM Tris/1mM MgCl_2_ (Sigma Aldrich, Zwijndrecht, Netherlands) at 37°C for 24 h. This was followed by washing in PBS (Sigma Aldrich, Zwijndrecht, Netherlands) + 2.7 mM EDTA for 3x1 h and overnight in ultrapure water. The next day, ACLs were washed in 1% Triton X-100 in Tris-EDTA (pH 7.6) for 72 h, washed in ultrapure water for 2 min, and again digested with Benzonase for 48 h under the conditions previously mentioned. ACLs were finally washed in PBS + 2.7 mM EDTA for 3x1 h and for 72 h in ultrapure water. Samples were then handled either according to the DNeasy Blood & Tissue kit (Qiagen, Venlo, Netherlands), or digested overnight at 60°C using 140 mg/ml papain (Sigma Aldrich, Zwijndrecht, Netherlands) in 100 mM phosphate buffer, 5 mM L-cysteine, 5 mM EDTA prior to DNA quantification using the Qubit platform (Invitrogen, Fisher Scientific, Landsmeer, Netherlands) or Nanodrop (Isogen Life Science, Utrecht, Netherlands) following the manufacturer’s instructions. Statistical differences were investigated using R (version 3.6.3).

**References**

1. Dede Eren, A. *et al.* Decellularized Porcine Achilles Tendon Induces Anti-inflammatory Macrophage Phenotype In Vitro and Tendon Repair In Vivo. *J. Immunol. Regen. Med.* **8**, 100027 (2020).
